# Association of Dynamic Changes in Peripheral Blood Indexes With Response to PD-1 Inhibitor-Based Combination Therapy and Survival Among Patients With Advanced Non-Small Cell Lung Cancer

**DOI:** 10.3389/fimmu.2021.672271

**Published:** 2021-05-14

**Authors:** Yuzhong Chen, Shaodi Wen, Jingwei Xia, Xiaoyue Du, Yuan Wu, Banzhou Pan, Wei Zhu, Bo Shen

**Affiliations:** ^1^ The Affiliated Cancer Hospital of Nanjing Medical University, Jiangsu Cancer Hospital & Jiangsu Institute of Cancer Research, Nanjing, China; ^2^ Key Hematological of Medical Science and Hematological Medicine of Jiangsu Province, School of Medicine, Jiangsu University, Zhenjiang, China

**Keywords:** non-small cell lung cancer, PD-1 inhibitor, combination therapy, carcinoembryonic antigen, neutrophil-to-lymphocyte ratio, neuron-specific enolase

## Abstract

**Background:**

PD-1 inhibitors have been routinely used in the treatment of advanced non-small cell lung cancer (NSCLC), and have demonstrated to significantly improve survivorship when combining with other conventional therapies, such as chemotherapy and anti-angiogenesis therapy. PD-L1 is the most commonly used biomarker to select benefiting groups, while not all patients with high PD-L1 expression benefit from immunotherapy. Therefore, identifying other prognostic and predictive biomarkers, including peripheral blood indexes, is essential.

**Methods:**

We retrospectively collected medical records and hematological data of 151 patients with advanced NSCLC treated with PD-1 inhibitor-based combination therapy in our hospital. The peripheral blood indexes of interest were NLR, PLR, PAR, Hb, LDH, CEA, and NSE. The association between peripheral blood indexes and treatment responses or survival outcomes was examined by multivariable logistic regression and Cox regression, respectively.

**Results:**

The decreased CEA at week 6 (OR = 4.209, 95%CI: 1.287-13.758) or 12 (OR = 7.267, 95%CI: 1.508-35.006) post-treatment was related to a higher disease control rate. The decrease or NLR at week 6 (OR = 3.081, 95%CI: 1.464-6.483) or 12 (OR = 3.304, 95%CI: 1.560-7.001) post-treatment, or CEA at week 12 post-treatment (OR = 2.469, 95%CI: 1.134-5.375), was associated with a higher objective response rate. Patients whose NLR (HR = 0.610, 95%CI: 0.411-0.907) or CEA (HR = 0.477, 95%CI: 0.320-0.710) decreased at week 6 post-treatment tended to have longer progression-free survival, and similar results were found in those with decreased NLR (HR = 0.587, 95%CI: 0.388-0.886) or CEA (HR = 0.406, 95%CI: 0.270-0.609) at week 12 post-treatment. Patients whose CEA (HR = 0.543, 95%CI: 0.339-0.871) or NSE (HR = 0.619, 95%CI: 0.386-0.994) decreased after 6 weeks post-treatment appeared to have longer overall survival, and the same was found for those whoseCEA (HR = 0.620, 95%CI: 0.390-0.986) or NSE (HR = 0.578, 95%CI: 0.353-0.947) was decreased at 12 weeks after treatment.

**Conclusion:**

Post-treatment NLR, CEA and NSE changes are suggestive indicators for the prognosis of NSCLC patients after immunotherapy.

## Introduction

Immunotherapy, especially immune checkpoint inhibitors (ICIs) represented by programmed cell death protein-1 (PD-1) inhibitors, has significantly improved the prognosis of patients with advanced non-small cell lung cancer (NSCLC). ICIs as a single agent or in combination with other treatments, such as chemotherapy and anti-angiogenic therapy, have become the standard treatment for driver gene-negative advanced NSCLC. However, approximately 50% of the patients showed no benefit from immunotherapy, and a proportion of them even experienced hyper-progression or fatal toxicity (1–4). Precise and reliable biomarkers are critical for identifying the patients who can potentially benefit from immunotherapy. Currently, programmed death-ligand 1 (PD-L1) and tumor mutation burden (TMB) remain as the most common biomarkers that are approved by the Food and Drug Administration (FDA) for predicting the efficacy of immunotherapy in NSCLC. Nevertheless, a certain percentage of patients with negative PD-L1 expression or low TMB can still benefit from immunotherapy. Oncogenic alterations, such as epidermal growth factor receptor (EGFR) and anaplastic lymphoma kinase (ALK), are usually associated with poor treatment response. Other potential predictive biomarkers, including microbiome, tumor infiltrating lymphocytes, gene signatures, multi-omics, etc. (5) are expensive, time-consuming for operation, and only tested on tissue specimens, which limit their clinical applications. Therefore, developing inexpensive and efficient biomarkers to select populations that can benefit from immunotherapy is urgently required.

In recent years, peripheral blood biomarkers representing tumor burden or inflammation have been increasingly studied for the purpose of predicting NSCLC treatment effect. The lower pre-treatment level or post-treatment declination of those biomarkers was previously shown to associate with the higher response rate and better prognosis in the patients (6–15). However, most studies were based on monotherapy, and the meaningfulness of baseline values remains controversial. Therefore, we conducted a retrospective study to further validate peripheral blood markers in predicting outcome and prognosis in patients with advanced NSCLC treated with PD-1 inhibitors-based combination therapy. The findings can serve as a designing reference for future stratified randomized controlled trials. In addition to the patient’s baseline clinical characteristics, the explored peripheral blood markers are as follows: neutrophil-to-lymphocyte ratio (NLR), platelet-to-lymphocyte ratio (PLR), platelet-to-albumin ratio (PAR), hemoglobin (Hb), lactate dehydrogenase (LDH), carcinoembryonic antigen (CEA) and neuron-specific enolase (NSE).

## Materials and Methods

### Study Design

We retrospectively identified and included 151 patients with advanced or relapsed NSCLC who received anti-PD-1-based combination therapy (pembrolizumab, sintilimab or toripalimab) at the Affiliated Cancer Hospital of Nanjing Medical University, China, from August 2018 to December 2019. Patients received 200mg pembrolizumab, 200mg sintilimab, or 240mg toripalimab intravenously once every 3 weeks. Combination chemotherapy was all based on platinum doublet chemotherapy, while the other drugs, including pemetrexed, docetaxel, paclitaxel/nab-paclitaxel, and gemcitabine was according to tumor histology. Bevacizumab was used as the combined anti-angiogenic drug. Patients who had inflammation or used steroids within 1 month were excluded.

Clinicopathological features of the patients, including age at the time of treatment, gender, pathology type, stage, Eastern Cooperative Oncology Group performance status (ECOG PS) score, smoking history, number of distant metastases, type of driver mutation, degree of tumor differentiation, treatment regimen, number of treatment lines, whether or not had received radiotherapy, and best response to treatment, were collected through electronic medical records or telephone follow-up. The peripheral blood indexes including NLR (absolute neutrophil count/absolute lymphocyte count), PLR (absolute platelet count/absolute lymphocyte count), PAR (absolute platelet count/albumin), hemoglobin (Hb), lactate dehydrogenase (LDH), and carcinoembryonic antigen (CEA), were collected before treatment, and 6 and 12 weeks after treatment (_0w_, _6w_, and _12w_). All of the abovementioned indexes were considered as binary variables in the analysis: the first four were dichotomized based on the median value, and the last three were dichotomized from the upper or lower limit of hematological tests based on clinical significance. Whole-body computed tomography scans were performed every 6-8 weeks after treatment to assess patients’ response to treatment according to The Response Evaluation Criteria in Solid Tumors (16). The last follow-up date was December 10^th^, 2020.

Treatment response was evaluated by objective response rate (ORR) and disease control rate (DCR), while survival was evaluated by progression-free survival (PFS) and overall survival (OS). Specifically, ORR was defined as the sum of complete response (CR) and partial response (PR), and DCR was defined as the sum of CR, PR, and stable disease (SD). PFS was defined as the time from initial treatment to clinical or imaging progression or death, and OS was defined as the time from initial treatment to the last follow-up or death, whichever came first.

This study was approved by the Institutional Review Board of Jiangsu Cancer Hospital. Patient’s informed consent was not necessary because this study was a retrospective study.

### Statistical Analysis

The baseline characteristics, peripheral blood indexes, and treatment response of the patients were reported as medians and interquartile ranges (IQRs) for continuous variables, or frequencies and percentages for categorical variables. For the response to treatment, we performed Chi-squared test (or Fisher’s exact test if the expected frequency of any cell in the contingency table is smaller than 5) to compare the distributions of the clinical factors/peripheral blood indexes between patients with and without the best response. We explored the relationship between clinical factors/peripheral blood indexes and best treatment response through multivariable logistic regression. For the survival outcomes, we used the Kaplan–Meier method to generate the PFS and OS survival curves, and the Log-rank test to compare survival outcomes among patients separated by the factors of interest. Then, we constructed the Cox proportional hazards model to estimate the association between clinical factors/peripheral blood indexes and survival outcomes, and used concordance index (C-index) to evaluate the discriminative ability of models, with closer to 1.0 indicating a better ability to correctly discriminate the outcome. Variables included in the multivariable analysis were selected based on clinical relevance and statistical significance in the Chi-square test or univariable analysis (*P* < 0.10). The above analysis was carried out for data obtained at 3 different time points. *P* < 0.05 was considered statistically significant. All tests were two-sided. SPSS 25.0 and GraphPad Prism 9.0.0 were used for data analysis and graphing.

## Results

### Patient Characteristics

A total of 151 patients were included in this study. **Table 1** shows detailed baseline clinicopathological characteristics. The median age of the patients was 63 years (IQR: 54-69), 76.2% of patients were male, 60.3% were smokers, and almost all patients were ECOG PS score 0-1, only four had PS 2. Most patients had distant metastasis (78.8%) and low degree of differentiation (76.8%). Among the 151 patients, 105 received anti-PD1 therapy combined with chemotherapy, 18 received anti-PD1 therapy combined with anti-angiogenesis therapy, and 28 received both; 61 patients were untreated, while 90 patients were retreated (≥ 2). More than half of the patients received radiotherapy during immunotherapy. Their detailed peripheral blood indexes are shown in **Table 2** and **Supplementary Tables 1** and **2**. The median follow-up time was 20.4 months (95%CI: 14.5-26.3).

**Table 1:** Patients’ characteristics at baseline and treatment response.

Characteristics	No. of patients(N = 151)	Percentage(%)
Age(years), median(IQR)	63(54-69)	
≥ 63	81	53.6
< 63	70	46.4
Gender		
Female	36	23.8
Male	115	76.2
Tumor histology		
Squamous	50	33.1
Non-Squamous	101	66.9
Adenocarcinoma	92	60.9
Others^†^	9	6.0
Stage		
Recurrence	29	19.2
Advanced	122	80.8
IIIB	29	19.2
IV	93	61.6
ECOG PS		
0-1	147	97.4
2	4	2.6
Smoking history		
Never	60	39.7
Now/ever	91	60.3
No. of metastasis sites		
0	32	21.2
1	72	47.4
2	33	21.9
≥ 3	14	9.3
Mutation type^‡^		
EGFR	28	18.5
KRAS	7	4.6
Wild-type	116	76.8
Degree of differentiation		
Low	116	76
Moderate/high	35	23.2
PD-1 inhibitor type		
Pembrolizumab	70	46.4
Sintilimab	66	43.7
Toripalimab	15	9.9
Combination regimen		
Chemotherapy	105	69.5
Anti-angiogenic therapy	18	11.9
Both	28	18.5
Lines of therapy		
1	61	40.4
2	49	32.5
≥ 3	41	27.2
Radiotherapy		
No	85	56.3
Yes	66	43.7
Best response		
CR	0	0.0
PR	46	30.5
SD	88	58.3
PD	17	11.3

^†^adenosquamouscarcinoma(n = 3), Sarcomatoid carcinoma(n = 2), otherwise(n = 4).

^‡^ALK mutation(n = 0).

ECOG, Eastern Cooperative Oncology Group; PS, performance status.

**Table 2:** Patients’ peripheral blood indexes before treatment (0 week).

Observation indexes	No. of patients(N = 151)	Percentage(%)
NLR, median(IQR)	2.96(2.13-4.54)	
> 2.96	75	49.7
≤ 2.96	76	50.3
PLR, median(IQR)	158.62(115.89-229.23)	
> 159	75	49.7
≤ 159	76	50.3
PAR(*10^9), median(IQR)	5.14 (3.98-6.42)	
≥ 5.15	75	49.7
< 5.15	76	50.3
Hb(g/L), median(IQR)	129.00(118.00-140.00)	
≥ 130	75	49.7
< 130	76	50.3
LDH(U/L), median(IQR)	206.00(181.00-258.00)	
> 245	42	27.8
≤ 245	109	72.2
CEA(ng/ml), median(IQR)	4.88(2.61-17.23)	
> 3.5	97	64.2
≤ 3.5	54	35.8
NSE^†^ (ng/ml), median(IQR)	16.47(13.08-22.13)	
> 16.3	72	50.7
≤ 16.3	70	49.3

^†^N = 142.

NLR, neutrophil-to-lymphocyte ratio; PLR, platelet-to-lymphocyte ratio; PAR, platelet-to-albumin ratio; Hb, hemoglobin; LDH, lactate dehydrogenase; CEA, carcinoembryonic antigen; NSE, neuron-specific enolase.

At the time of administrative censoring (Dec 10^th^, 2020), 83 patients have died, 48 were still receiving immunotherapy, 20 lost to follow-up or discontinued the therapy due to toxicity. Five patients failed to reach the fourth cycle of medication. No patient reached CR, 46 (30.5%) reached PR, 88 (58.3%) reached SD, and 17 (11.3%) reached “progressive disease (PD)”. The ORR and DCR were 30.5% and 88.7%, respectively. Median PFS and OS were 8.0 (95%CI: 7.2-8.8) and 15.3 months (95%CI: 13.8-16.8), respectively (**Supplementary Figure 1**).

### Associations Between PD-1 Inhibitor Type/Combination Regimen and Treatment Response/Survival Outcomes

We conducted a univariate analysis of PD-1 inhibitor type and combination regimen while no differences in DCR, ORR, PFS and OS were found(**Supplementary Table 3**).

### Associations Between Clinical Factors/Peripheral Blood Indexes and Treatment Response

**Figure 1:**
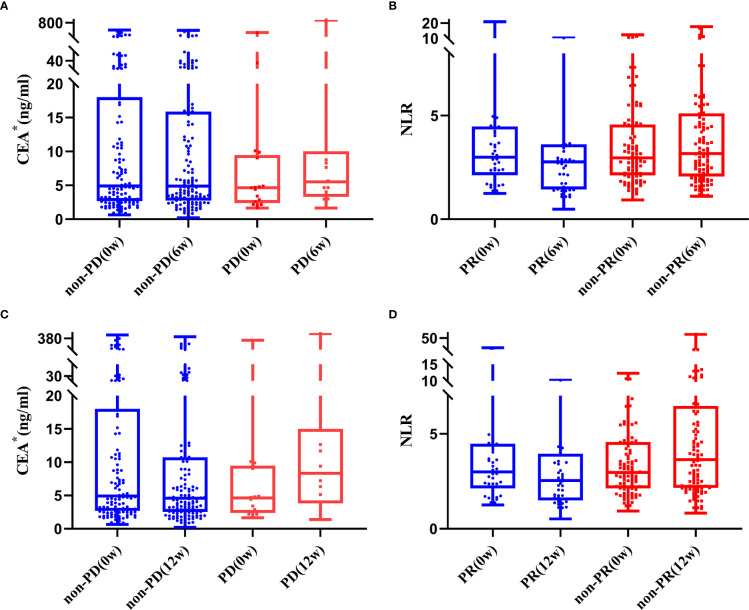
Trend of CEA **(A, B)** and NLR **(C, D)** in patients with and without response to treatment (*one extreme value was removed).

As shown in **Supplementary Table 4**, age, lines of therapy, radiotherapy, LDH_0w_, NLR_6w_, LDH_6w_, CER_6w_, NLR_12w_ and CEA_12w_ was associated with DCR, while age, radiotherapy, Hb_0w_, CEA_0w_, NLR_6w_, LDH_6w_, NLR_12w_, LDH_12w_ and CEA_12w_ were associated with ORR, without any adjustment. Considering that most of the patients received no radiotherapy at the early stage of treatment, “radiotherapy” was not included in the multivariable logistic regression analysis. Age ≤ 63 years (OR = 3.103, 95%CI: 1.035-9.306), CEA_6w_ Down (OR = 4.209, 95%CI: 1.287-13.758), and CEA_12w_ Down (OR = 7.267, 95%CI: 1.508-35.006) were significantly associated with higher DCR, while NLR_12w_ Down (OR = 4.682, 95%CI: 0.962-22.796) had marginal significance (*P* = 0.056). Age ≤ 63 years (OR = 2.273, 95%CI: 1.100-4.697), NLR_6w_ Down (OR = 3.081, 95%CI: 1.464-6.483), NLR_12w_ Down (OR = 3.304, 95%CI: 1.560-7.001) and CEA_12w_ Down (OR = 2.469, 95%CI: 1.134-5.375) were significantly associated with higher ORR (**Table 3** and **Figure 1**).

**Table 3:** Multivariable Logistic regression models for DCR and ORR.

		Disease control rate				Objective response rate		
		Odds ratio	95%CI	*P*		Odds ratio	95%CI	*P*
0w	Age (years)				Age (years)			
	> 63	1			> 63	1		
	≤ 63	3.103	1.035-9.306	0.043	≤ 63	2.273	1.100-4.697	0.027
6w	Age (years)				Age (years)			
	> 63	1	0.976-9.162	0.055	> 63	1	1.122-5.028	0.024
	≤ 63	2.991			≤ 63	2.375		
	CEA				NLR			
	Up	1	1.287-13.758		Up	1	1.464-6.483	0.003
	Down	4.209		0.017	Down	3.081		
12w	NLR				NLR			
	Up	1	0.962-22.796	0.056	Up	1	1.560-7.001	0.002
	Down	4.682			Down	3.304		
	CEA				CEA			
	Up	1	1.		Up	1		0.023
	Down	7.267	508-35.006	0.013	Down	2.469	1.134-5.375	

NLR, neutrophil-to-lymphocyte ratio; CEA, carcinoembryonic antigen.

### Associations Between Clinical Factors/Peripheral Blood Indexes and Survival Outcomes

**Figure 2:**
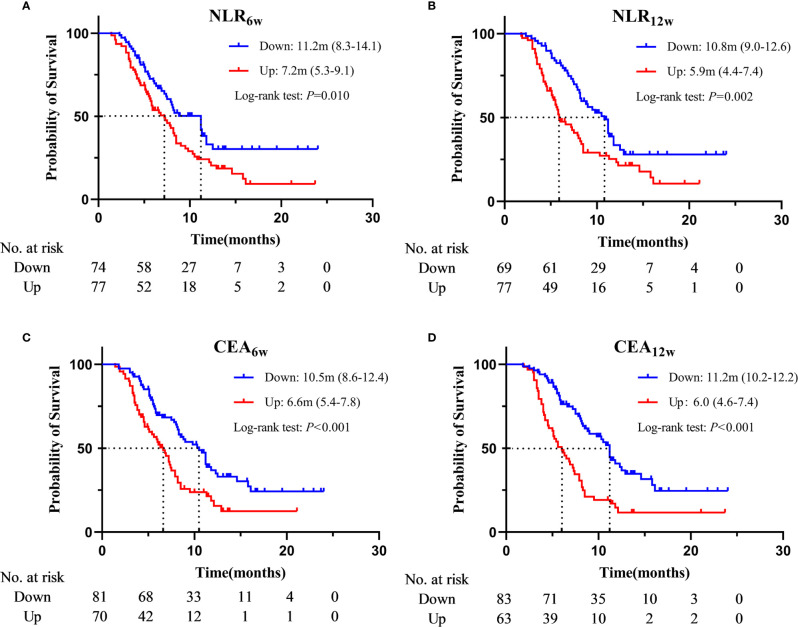
Kaplan–Meier curves for PFS according to NLR_6w_ **(A)**, NLR_12w_ **(B)**, CEA_6w_ **(C)** and CEA_12w_ **(D)**.

**Figure 3:**
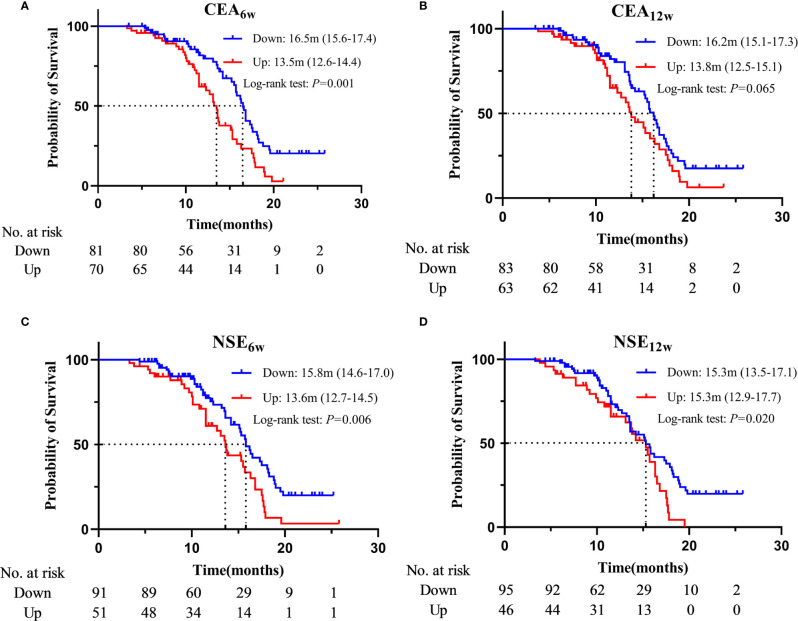
Kaplan–Meier curves for OS according to CEA_6w_ **(A)**, CEA_12w_ **(B)**, NSE_6w_ **(C)** and NSE_12w_ **(D)**.

Based on the Cox regression analysis (**Tables 4**, **5**, only significant variables from univariable analysis are shown), we found that ECOG PS (0 vs. 2: 11.2m vs. 4.1m, HR = 0.160, 95%CI: 0.050-0.515; 1 vs. 2: 7.9m vs. 4.1m, HR = 0.217, 95%CI: 0.077-0.612), radiotherapy (Yes vs. No: 11.2m vs. 7.2m, HR = 0.536, 95%CI: 0.359-0.800), NLR_6w_ (Down vs. Up: 11.2m vs. 7.2m, HR = 0.610, 95%CI: 0.411-0.907), CEA_6w_ (Down vs. Up: 10.5m vs. 6.6m, HR = 0.477, 95%CI: 0.320-0.710), NLR_12w_ (Down vs. Up: 10.8m vs. 5.9m, HR = 0.587, 95%CI: 0.388-0.886) and CEA_12w_ (Down vs. Up: 11.2m vs. 6.0m, HR = 0.406, 95%CI: 0.270-0.609) were independently associated with PFS (**Figure 2**). While CEA_0w_ (>3.5ng/ml vs. ≤ 3.5 ng/ml: 15.8m vs. 13.6m, HR = 0.611, 95%CI: 0.388-0.963), CEA_6w_ (Down vs. Up: 16.5m vs. 13.5m, HR = 0.543, 95%CI: 0.339-0.871), NSE_6w_ (Down vs. Up: 15.8m vs. 13.6m, HR = 0.619, 95%CI: 0.386-0.994), CEA_12w_ (Down vs. Up: 16.2m vs. 13.8m, HR = 0.620, 95%CI: 0.390-0.986) and NSE_12w_ (Down vs. Up: 15.3m vs. 15.3m, HR = 0.578, 95%CI: 0.353-0.947) were independently associated with OS (**Figure 3**). The C-index of model_6w_ and model_12w_ to predict PFS was 0.673 (95% CI: 0.623-0.724) and 0.691 (95% CI: 0.642-0.739), respectively. While the C-index of model_6w_ and model_12w_ to predict OS was 0.615 (95% CI: 0.553-0.677) and 0.614 (95% CI: 0.542-0.686), respectively.

**Table 4:** Univariable Cox regression analysis for PFS and OS.

	Progression free survival				Overall survival		
	unadjusted HR	95%CI	*P*		unadjusted HR	95%CI	*P*
ECOG PS				Stage			
2	1			Recurrence	1		
0	0.165	0.051-0.528	0.002	IIIB	1.747	0.960-3.178	0.068
1	0.240	0.085-0.673	0.007	IV	1.153	0.678-1.961	0.599
NLR_6w_				Mutation type			
Up	1			Wild-type	1		
Down	0.604	0.408-0.894	0.012	EGFR	1.779	0.993-3.189	0.053
Hb_6w_				KRAS	1.145	0.416-3.146	0.793
Up	1			PLR_0w_			
Down	0.682	0.446-1.045	0.079	Up	1		
CEA_6w_				Down	0.679	0.439-1.052	0.083
Up	1			CEA_0w_			
Down	0.510	0.344-0.756	0.001	Up	1		
NLR_12w_				Down	0.620	0.393-0.977	0.039
Up	1			CEA_6w_			
Down	0.536	0.357-0.802	0.002	Up	1		
CEA_12w_				Down	0.497	0.320-0.771	0.002
Up	1			NSE_6w_			
Down	0.413	0.276-0.618	0.000	Up	1		0.007
Radiotherapy				Down	0.537	0.340-0.846	
Up	1			CEA_12w_			
Down	0.536	0.359-0.799	0.002	Up	1		
				Down	0.662	0.424-1.033	0.069
				NSE_12w_			
				Up	1		
				Down	0.573	0.356-0.923	0.022

ECOG, Eastern Cooperative Oncology Group; PS, performance status; NLR, neutrophil-to-lymphocyte ratio; PLR, platelet-to-lymphocyte ratio; Hb, hemoglobin; CEA, carcinoembryonic antigen; NSE, neuron-specific enolase.

**Table 5:** Multivariable Cox regression analysis for PFS and OS.

		Progression free survival				Overall survival		
		adjusted HR	95%CI	*P*		adjusted HR	95%CI	*P*
0w	ECOG PS				CEA(ng/ml)			
	2	1			≤ 3.5	1		
	0	0.160	0.050-0.515	0.002	> 3.5	0.611	0.388-0.963	0.034
	1	0.217	0.077-0.612	0.004				
	Radiotherapy							
	No	1						
	Yes	0.536	0.359-0.800	0.002				
6w	ECOG PS				CEA			
	2	1			Up	1		
	0	0.126	0.038-0.415	0.001	Down	0.543	0.339-0.871	0.011
	1	0.207	0.072-0.592	0.003	NSE			
	Radiotherapy				Up	1		
	No	1			Down	0.619	0.386-0.994	0.047
	Yes	0.562	0.375-0.841	0.005				
	NLR							
	Up	1						
	Down	0.610	0.411-0.907	0.015				
	CEA							
	Up	1						
	Down	0.477	0.320-0.710	0.000				
12w	ECOG PS				CEA			
	2	1			Up	1		0.043
	0	0.129	0.038-0.437	0.001	Down	0.620	0.390-0.986	
	1	0.224	0.077-0.649	0.006	NSE			
	Radiotherapy				Up	1		0.029
	No	1			Down	0.578	0.353-0.947	
	Yes	0.569	0.375-0.865	0.008				
	NLR							
	Up	1						
	Down	0.587	0.388-0.886	0.011				
	CEA							
	Up	1						
	Down	0.406	0.270-0.609	0.000				

ECOG, Eastern Cooperative Oncology Group; PS, performance status; NLR, neutrophil-to-lymphocyte ratio; CEA, carcinoembryonic antigen; NSE, neuron-specific enolase.

**Figure 4:**
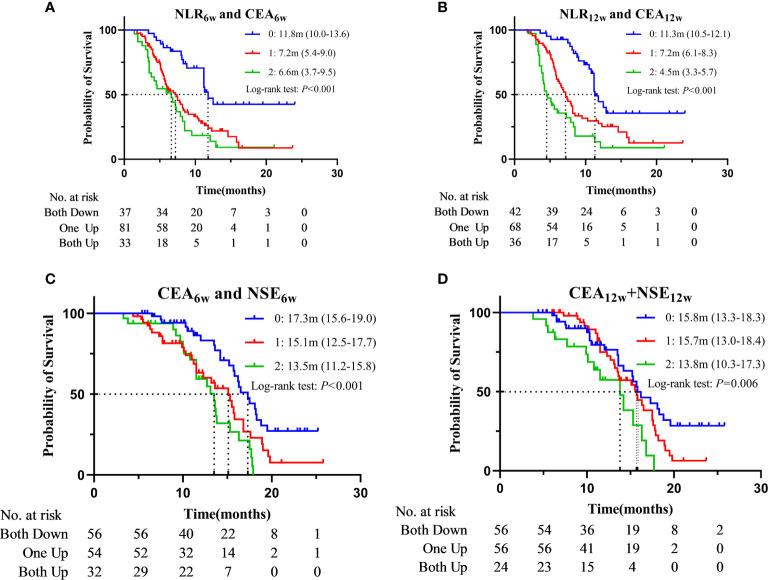
Kaplan–Meier curves for PFS according to “NLR_6w_ and CEA_6w_” **(A)** or “NLR_12w_ and CEA_12w_” **(B)** and for OS according to “CEA_6w_ and NSE_6w_” **(C)** or “CEA_12w_ and NSE_12w_” **(D)** (0: Both Down, 1: One Up, 2: Both Up).

Then, we grouped the patients according to the dynamic changes of NLR + CEA and CEA + NSE, and constructed the corresponding survival curve (**Figure 4** and **Supplementary Figure 3**). Patients with decreased NLR and CEA at week 6 post-treatment (median: 11.8m, 95%CI: 10.0-13.6) had a significantly longer PFS than those with increases in single (median: 7.2m, 95%CI: 5.4-9.0, *P* < 0.001) or both (median: 6.6m, 95%CI: 3.7-9.5, *P* < 0.001) indicators, while PFS was only numerically prolonged in “patients One up” compared to that in “patients Both up” (*P* = 0.172). Similarly, compared with the patients with increases in single (median: 7.2m, 95%CI: 6.1-8.3, *P* < 0.001) or both (median: 4.5m, 95%CI: 3.3-5.7, *P* < 0.001), those with decreased NLR and CEA at week 12 post-treatment (median: 11.3m, 95%CI: 10.5-12.1) had a significantly longer PFS, and “patients One up” had a significantly longer PFS than “patients Both up” (*P* = 0.015). Patients with decreased CEA and NSE at week 6 post-treatment (median: 17.3m, 95%CI: 15.1-19.0) had a significantly longer OS than those with increases in single (median: 15.1m, 95%CI: 12.5-17.7, *P* = 0.012) or both (median: 13.5m, 95%CI: 11.2-15.8, *P* < 0.001), while the latter two groups of patients had only numerical differences in OS (*P* = 0.166). Similarly, compared to the patients with increased CEA and NSE at week 12 post-treatment (median: 13.8m, 95%CI: 10.3-17.3), those with decreases in single (median: 15.7m, 95%CI: 13.0-18.4, *P* = 0.015) or both (median: 15.8m, 95%CI: 13.3-18.3, P = 0.006) indicators had a significantly longer OS, while the latter two groups of patients had only numerical difference in OS (*P* = 0.139). We further divided all the patients into 4 groups according to CEA_0w_ (ng/ml) and CEA_6w_: ≤ 3.5 and Down, ≤ 3.5 and Up, > 3.5 and Down, and > 3.5 and Up. The median OSs were 13.6m (13.2-14.0), 13.2m (11.4-15.0), 16.5m (15.5-17.5) and 13.6m (12.2-15.0), respectively. Pairwise comparison revealed that the OS of the third group (> 3.5 and Down) was significantly longer than that of the second group (≤ 3.5 and Up) (*P* = 0.005).

## Discussion

Compared with conventional chemotherapy, ICIs are highly effective on advanced NSCLC, especially in patients with PD-L1 TPS≥50%. However, ICIs alone may not be the best option for patients with PD-L1 TPS between 1-49% (1, 2). In addition, several studies on ICIs-based combination therapy have demonstrated its significant benefits regardless of PD-L1 expression status (3, 4, 17), without inducing concomitant side effects and financial burden for the patients. Efficient, convenient and inexpensive markers are needed to help characterize the patients who can potentially benefit from the ICIs treatment. To our knowledge, this is the first study to assess the association between peripheral blood markers and the outcome of PD-1 inhibitor-based combination therapy in a Chinese population, and our data can provide the basis for stratification in later RCTs. In our study, we found that the dynamic changes of NLR, CEA and NSE after treatment were strongly associated with the treatment outcomes and prognosis of the patients. Briefly, patients with decreased NLR or CEA at 6 or 12 weeks after treatment had better efficacy and longer PFS, and those with decreased CEA or NSE had longer OS.

Inflammation not only is critical at various stages of tumor development and progression, but also can either positively or negatively influence tumor immune-surveillance and therapeutic response. Therefore, inflammatory markers could be potential prognostic factors in immunotherapy for NSCLC. During the treatment of anti-PD-1/PD-L1 antibodies, the activation of lymphocytes is necessary for restoring the anti-tumor immune response (18). Such an immune response is the result of multiple interactions between T cells and other regulatory cells, including neutrophils, which play a leading role in the immune environment of NSCLC. Tumor-associated neutrophils have diametrically opposite effects at different molecular levels, which can be divided as anti-tumor N1 type and tumor-promoting N2 type (19–21). A prospective study with 104 patients identified a predictive immune signature (LIPS) based on the peripheral blood immunophenotype, including CD14^high^ monocytes, CD8^+^/PD-1^+^ T cells, plasmacytoid dendritic cells, neutrophils, and CD3^+^/CD56^+^/CD16^+^ natural killer T cells (22). Based on LIPS, patients were categorized into low- and high-risk groups, and both PFS and OS were significantly longer for patients in the low-risk group than in the high-risk group, regardless of PD-L1 expression. This suggests that increased neutrophils counts in the peripheral blood were associated with less benefit from ICI. Another retrospective cohort study with 1714 patients examined the association between pretreatment NLR and TMB levels and survival among patients treated with ICI, and found that the patients with NLR-high/TMB-low had the worst prognosis, while those in the NLR-low/TMB-high group had the best prognosis (23). Other studies also found that patients with advanced NSCLC receiving immunotherapy with high NLR or PLR value (i.e., markers of chronic inflammation) usually showed poor prognosis (24–27). The nutritional status of patients is usually related to their prognosis, and serum albumin is considered a sensitive indicator of general nutritional status. A retrospective cohort study found that PAR is a potential prognostic biomarker for patients who underwent complete surgical resection for NSCLC (28). However, we found no correlation between treatment response or prognosis and PLR or PAR, regardless of their baseline values or changes after treatment. Moreover, no predictive significance of baseline values of NLR was identified, while patients with decreased NLR after treatment (whether at week 6 or 12) were more likely to achieve PR and had longer PFS.

Other peripheral blood indexes have also been proposed to be potential predictors for the effect of ICIs treatment. A recent prospective cohort study showed that normal pre-treatment level of Hbis a favorable prognostic factor for ICIs treatment in patients with advanced NSCLC (7). Furthermore, previous studies have shown that baseline level of LDH, a biomarker related to tumor burden, was associated with the poor treatment outcomes in NSCLC patients, which can also predict the prognosis of the patients treated with ICIs (29–31). However, our study did not find any association between Hb or LDH and treatment efficacy or prognosis. Several studies have shown that repeated measurements of serum tumor markers in patients with advanced NSCLC, especially CEA and NSE, may help clinicians assess the efficacy of anti-PD-1 monotherapy and predict the prognosis (8, 15, 32, 33), which supports our finding in the current study. Patients with decreased CEA after treatment had better outcomes and longer survival; CEA declination at week 6 and 12 post-treatment was associated with52.3% and 59.4% lower risk of tumor progression, respectively, and they were associated with 45.7% and 38.0% lower risk of death. These findings also reflect the significance of repeated measurements at different time points. However, prolongation of OS in patients with decreased NSE after treatment was identified, while it was not detected in those with decreased PFS. In addition, it is also noteworthy that the longer OS in patients with higher baseline CEA is due to that their CEA was decreased after treatment which may suggest that changes in indicators after treatment are more indicative than baseline values.

Recent studies suggest that the prognosis of immunotherapy is independent of age (34). We found better treatment outcomes in younger patients, while no significant difference in survival was observed between patients with different ages. Most immunotherapy-related RCTs excluded patients with ECOG PS ≥ 2, so data on selecting an effective population for immunotherapy based on ECOG PS status are scarce. Two retrospective studies demonstrated that NSCLC patients with ECOG PS ≥ 2 were not suitable for immunotherapy (35, 36), which is consistent with the results found in our study, though we only included 4 patients with PS = 2. The combination of radiotherapy and immunotherapy usually introduces synergistic treatment effects and increases the occurrence of the “abscopal effect” (37). Two previous clinical trials have confirmed that the combination of the two can significantly improve the efficiency of treatment (38, 39). In our study, patients who received radiotherapy during immunotherapy had better prognosis; their risk of progression was reduced by 43.1%, in comparison with those received no radiotherapy, though no differences in OS was observed.

In summary, we found that repeated measurements of NLR, CEA and NSE have greater value in assessing treatment efficacy and predicting prognosis, although the time point for repeated measurements cannot be fully determined. However, no significant results were found for the other parameters, attributed to: a). all the patients receive combined treatment, while chemotherapy only will affect these peripheral blood indexes; b). the best cut-off value was not optimal, which is currently uncertain; c). the follow-up time period was short, and several patients were lost because of COVID-2019. In addition, this study found a shorter median survival time than previous similar clinical trials, possibly due to that only 40.4% of the patients received initial treatment, and 48 patients still receive anti-PD-1 immunotherapy as of the follow-up time.

Our study has several limitations. First, it was a single-center retrospective study with a relatively small size, and Multi-center prospective cohort studies with larger sample sizes are warranted in the future. Second, the selection of peripheral blood indexes and time points for repeated measurement may have influence on the conclusion. Single indicators such as white blood cells, platelets, red blood cells and neutrophils were not included for analysis since most previous studies have shown that single blood parameters had no prognostic value; in addition C-reactive protein, cytokeratin-19-fragment and other tumor markers are not routinely tested in our institution. Moreover, the response to treatment may be reflected at various time points in different patients. In this study, 6 and 12 weeks’ time points were selected to investigate the potential association between peripheral blood indexes at the early time of treatment and the treatment efficacy and prognosis of the patients. Third, the changes of peripheral blood indexes might be induced by single chemotherapy; therefore, a positive control group with patients treated with chemotherapy only would be optimal. However, we were unable to enroll a sufficient number of patients in this group, since chemotherapy alone is rare for current clinical practice.

## Conclusion

In conclusion, in advanced NSCLC patients, the decreased NLR and CEA after treatment are independent predictors of their response to combined immunotherapy, while the decreased CEA and NSE are associated with a better prognosis.

## Data Availability Statement

The raw data supporting the conclusions of this article will be made available by the authors, without undue reservation.

## Ethics Statement

The study involving human participants was reviewed and approved by the Ethics Committee of Jiangsu Cancer Hospital. Patient’s informed consent was not necessary because this study was a retrospective study.

## Author Contributions

YC and BS: idea & design. YC, SW, JX and BP: data collection & collation. YC, SW, XD, YW and WZ: data analysis & interpretation. YC, SW and BS: manuscript writing. All authors contributed to the article and approved the submitted version.

## Funding

This work was supported by the National Natural Science Foundation of China (Grant No. 81972822, 81972313) and Wu Jieping Medical Foundation (Grant No.320.6750.19060).

## Conflict of Interest

The authors declare that the research was conducted in the absence of any commercial or financial relationships that could be construed as a potential conflict of interest.

## Supplementary Material

Supplementary Figure 1 Kaplan–Meier curves for PFS **(A)** and OS **(B)** in the overall population.

Supplementary Figure 2 Kaplan–Meier curves for PFS according to ECOG **(A)**, Radiotherapy **(B).**

Supplementary Figure 3 Kaplan–Meier curves for OS according to “CEA0w and CEA6w”.
