# Corrigendum: Association of Dynamic Changes in Peripheral Blood Indexes With Response to PD-1 Inhibitor-Based Combination Therapy and Survival Among Patients With Advanced Non-Small Cell Lung Cancer

**DOI:** 10.3389/fimmu.2021.713268

**Published:** 2021-06-07

**Authors:** Yuzhong Chen, Shaodi Wen, Jingwei Xia, Xiaoyue Du, Yuan Wu, Banzhou Pan, Wei Zhu, Bo Shen

**Affiliations:** ^1^ The Affiliated Cancer Hospital of Nanjing Medical University, Jiangsu Cancer Hospital & Jiangsu Institute of Cancer Research, Nanjing, China; ^2^ Key Hematological of Medical Science and Hematological Medicine of Jiangsu Province, School of Medicine, Jiangsu University, Zhenjiang, China

**Keywords:** non-small cell lung cancer, PD-1 inhibitor, combination therapy, carcinoembryonic antigen, neutrophil-to-lymphocyte ratio, neuron-specific enolase

In the original article, there was an error in the Ethics Statement: “The studies involving human participants were reviewed and approved by the Ethics Committee of the First Hospital of Shanxi Medical University. The patients/participants provided their written informed consent to participate in this study.”

A correction has been made:

“The study involving human participants was reviewed and approved by the Ethics Committee of Jiangsu Cancer Hospital. Patient’s informed consent was not necessary because this study was a retrospective study.”

The authors apologize for this error and state that this does not change the scientific conclusions of the article in any way. The original article has been updated.

