# Supplementary figures and images for: Association of Dynamic Changes in Peripheral Blood Indexes With Response to PD-1 Inhibitor-Based Combination Therapy and Survival Among Patients With Advanced Non-Small Cell Lung Cancer
Source: Front Immunol. 2021 May 14;12:672271. doi: 10.3389/fimmu.2021.672271 (PMC8161505; doi:10.3389/fimmu.2021.672271)

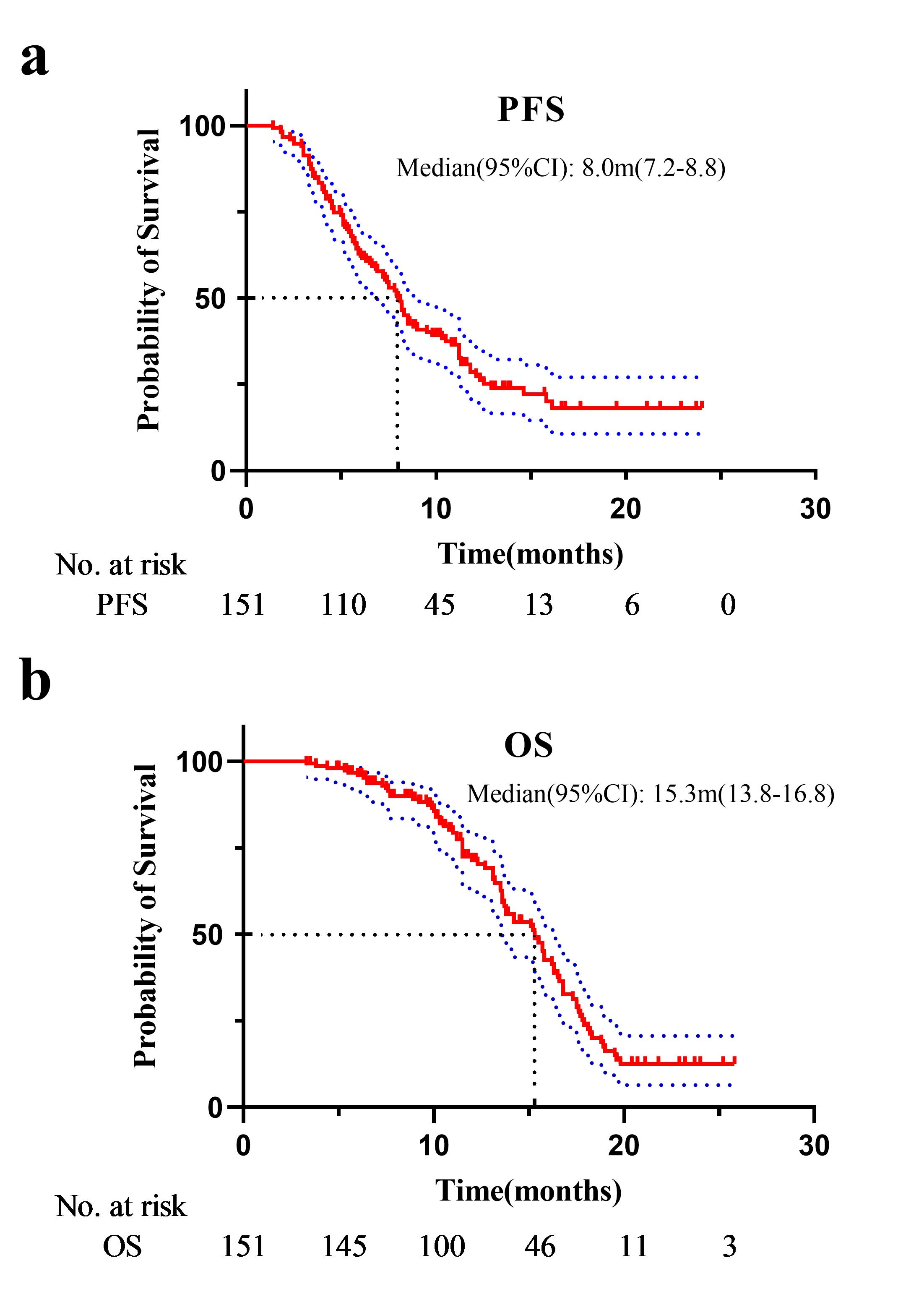

Supplement: Supplementary Figure 1 — Kaplan–Meier curves for PFS (A) and OS (B) in the overall population. [file Image_1.tif]

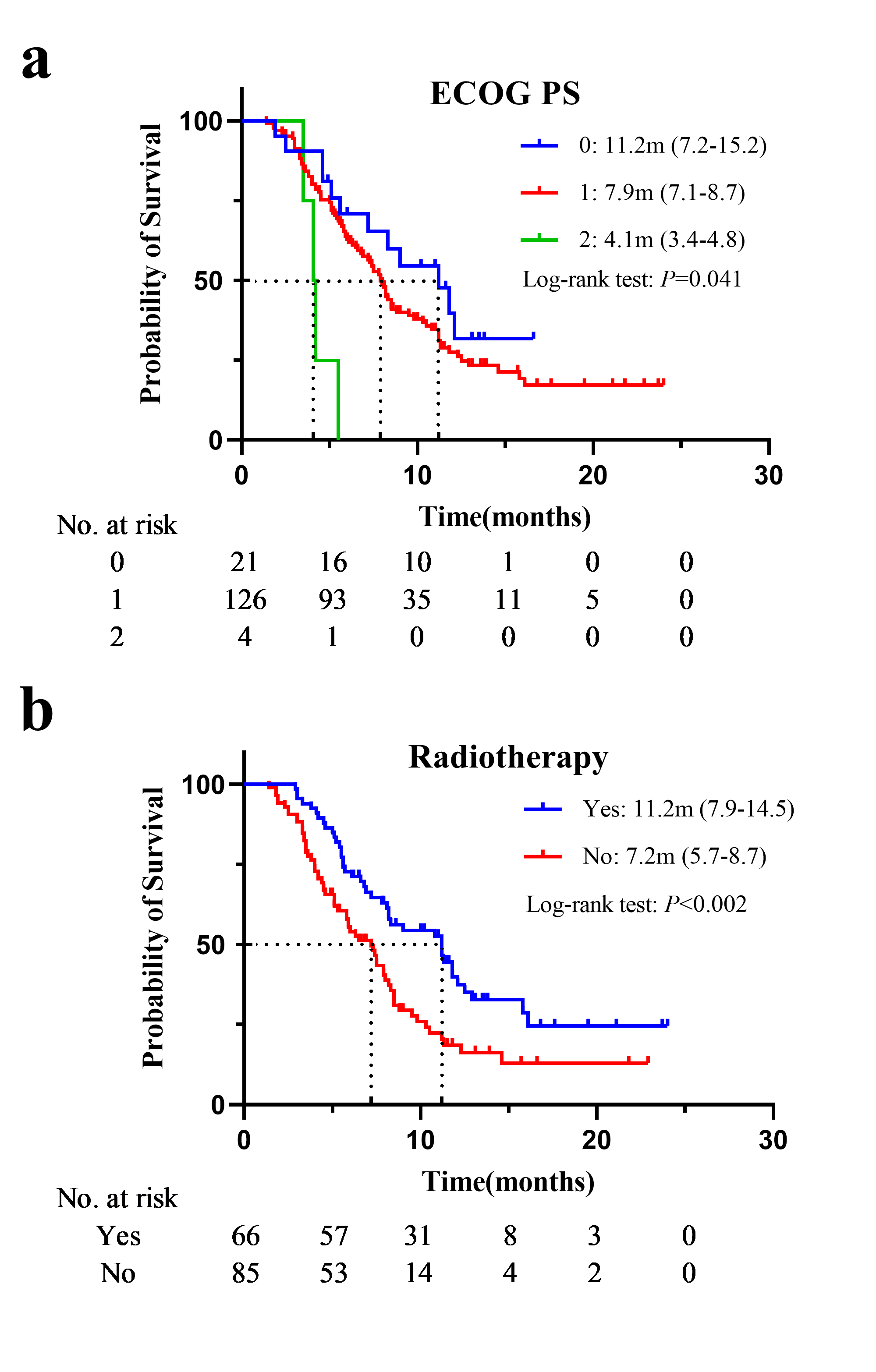

Supplement: Supplementary Figure 2 — Kaplan–Meier curves for PFS according to ECOG (A), Radiotherapy (B). [file Image_2.tif]

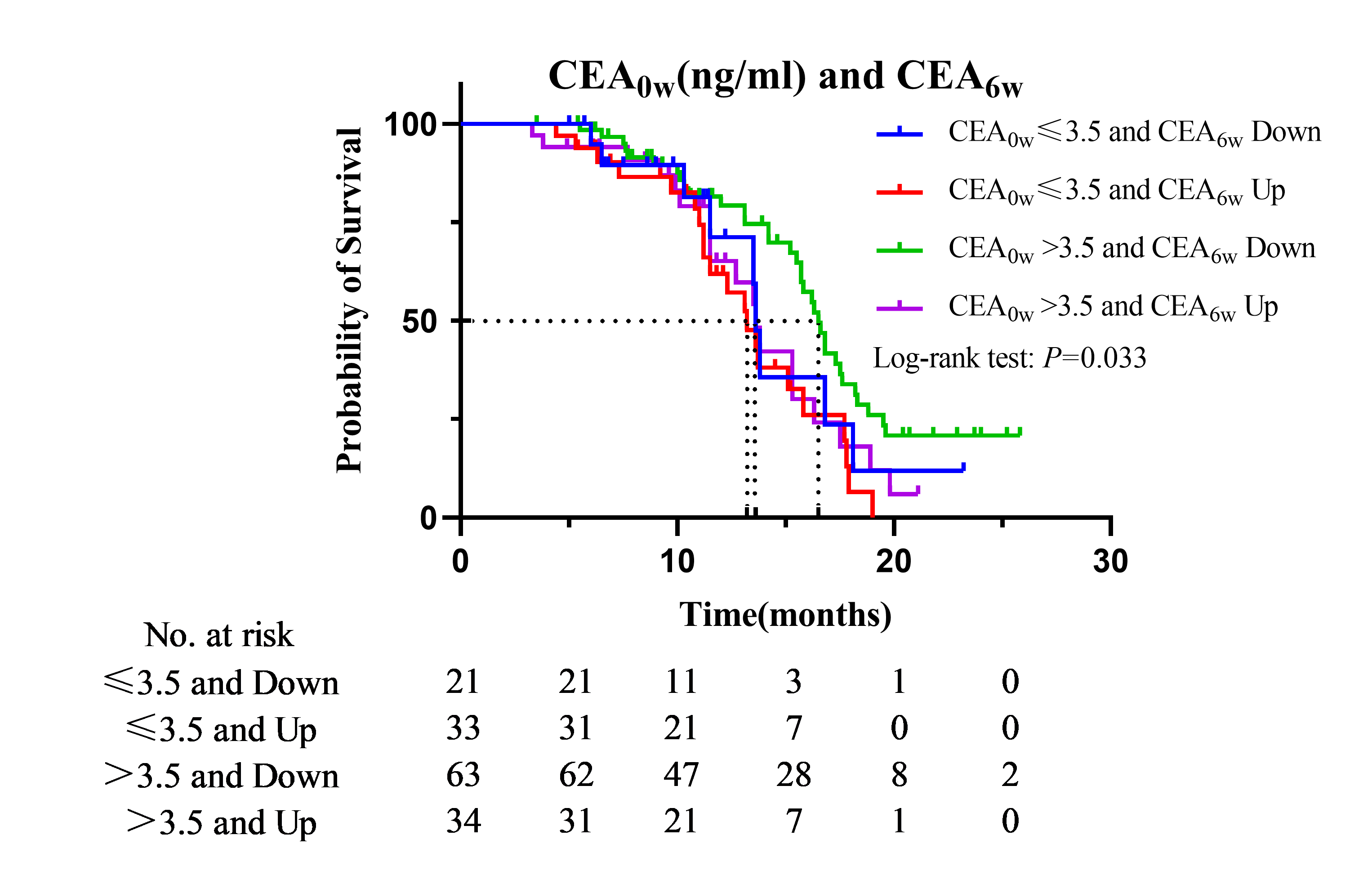

Supplement: Supplementary Figure 3 — Kaplan–Meier curves for OS according to “CEA0w and CEA6w”. [file Image_3.tif]
