# Supplementary material for: Association of Dynamic Changes in Peripheral Blood Indexes With Response to PD-1 Inhibitor-Based Combination Therapy and Survival Among Patients With Advanced Non-Small Cell Lung Cancer
Source: Front Immunol. 2021 May 14;12:672271. doi: 10.3389/fimmu.2021.672271 (PMC8161505; doi:10.3389/fimmu.2021.672271)
Supplement: Supplementary file 4 [file Table_1.docx]

**Suppl. Table 1** Patients’ peripheral blood indexes after treatment(6 weeks)

| Observation indexes | No. of patients(N=151) | Percentage(%) |
| --- | --- | --- |
| NLR, median(IQR) Up  Down | 2.88(1.94-4.56)  77  74 | 51.0  49.0 |
| PLR, median(IQR) Up  Down | 155.91(112.02-241.56)  71  80 | 47.0  53.0 |
| PAR(*10^9), median(IQR)  Up  Down | 4.75(3.78-6.42)  67  84 | 44.4  55.6 |
| Hb(g/L), median(IQR) Up  Down | 126.00(112.00-133.00)  49  102 | 32.5  67.5 |
| LDH(U/L), median(IQR)  Up  Down | 228.00(182.00-264.00)  81  70 | 53.6  46.4 |
| CEA(ng/ml), median(IQR)  Up  Down | 5.07(2.94-15.27)  97  54 | 64.2  35.8 |
| NSE^†^(ng/ml), median(IQR)  Up  Down | 14.71(11.72-18.88)  51  91 | 35.9  64.1 |

† N=142

Up: elevated compared to pre-treatment Down: decreased compared to pre-treatment

Abbreviations: NLR: neutrophil-to-lymphocyte ratio; PLR: platelet-to-lymphocyte ratio; PAR: platelet-to-albumin ratio; Hb: hemoglobin; LDH: lactate dehydrogenase; CEA: carcinoembryonic antigen; NSE: neuron-specific enolase

**Suppl. Table 2** Patients’ peripheral blood indexes after treatment(12 weeks)

| Observation indexes | No. of patients(N=146) | Percentage(%) |
| --- | --- | --- |
| NLR, median(IQR) Up  Down | 3.08(2.03-5.50)  77  69 | 52.7  47.3 |
| PLR, median(IQR) Up  Down | 153.22(102.26-244.57)  68  78 | 46.6  53.4 |
| PAR(*10^9), median(IQR)  Up  Down | 4.57 (3.46-6.27)  55  91 | 37.7  62.3 |
| Hb(g/L), median(IQR) Up  Down | 121.50(112.00-135.00)  48  98 | 31.8  64.9 |
| LDH(U/L), median(IQR)  Up  Down | 221.50 (185.50-273.00)  82  64 | 56.2  43.8 |
| CEA(ng/ml), median(IQR)  Up  Down | 4.66(2.58-11.47)  63  83 | 43.2  56.8 |
| NSE^†^(ng/ml), median(IQR)  Up  Down | 14.26(11.26-17.21)  46  95 | 32.6  67.4 |

† N=141

Up: elevated compared to pre-treatment Down: decreased compared to pre-treatment

Abbreviations: NLR: neutrophil-to-lymphocyte ratio; PLR: platelet-to-lymphocyte ratio; PAR: platelet-to-albumin ratio; Hb: hemoglobin; LDH: lactate dehydrogenase; CEA: carcinoembryonic antigen; NSE: neuron-specific enolase.

**Suppl. Table 3** Results of univariate analysis of PD-1 inhibitor type and combination regimen

|  | DCR^†^ | ORR^†^ | PFS^‡^ | OS^‡^ |
| --- | --- | --- | --- | --- |
| PD-1 inhibitor type |  |  |  |  |
| Toripalimab | 1 | 1 | 1 | 1 |
| Sintilimab | 0.711 (0.132-3.818) | 0.684 (0.174-2.692) | 1.341 (0.680-2.647) | 0.857 (0.437-1.683) |
| Pembrolizumab | 0.912 (0.173-4.810) | 0.427 (0.109-1.667) | 1.267 (0.632-2.542) | 1.250 (0.631-2.477) |
| Combination regimen |  |  |  |  |
| Both | 1 | 1 | 1 | 1 |
| Chemotherapy | 0.702 (0.205-2.400) | 0.955 (0.380-2.398) | 1.325 (0.781-2.245) | 1.022 (0.570-1.833) |
| Anti-angiogenic therapy | 0.750 (0.123-4.589) | 0.629 (0.180-2.201) | 1.334 (0.636-2.798) | 1.192 (0.528-2.688) |

† Odds ratio (95%CI)

‡ Hazard ratio (95%CI)

Abbreviations: DCR: Disease control rate; ORR: Objective response rate; PFS: progression free survival; OS: overall survival

**Suppl. Table 4** Associations of clinical factors and peripheral blood indexes with treatment response using chi-squared test or Fisher exact test

| Disease control rate | | | Objective response rate | |
| --- | --- | --- | --- | --- |
|  | n(%) | *P* | n(%) | *P* |
| Age(years) |  | 0.033 |  | 0.025 |
| ≥63  ＜63 | 76/81(93.8)  58/70(82.9) |  | 31/81(38.3)  15/70(21.4) | |
| Gender |  | 0.555 |  | 0.668 |
| Female | 31/36(86.1) |  | 12/36(33.3) | |
| Male | 103/115(89.6) |  | 34/115(29.6) | |
| Tumor histology |  | 0.373 |  | 0.644 |
| Squamous | 46/50(92.0) |  | 14/50(28.0) | |
| Non- Squamous | 88/101(87.1) |  | 32/101(31.7) | |
| Stage |  | 0.743 |  | 0.203 |
| Recurrence | 25/29(86.2) |  | 6/29(20.7) | |
| Advanced | 109/122(89.3) |  | 40/122(32.8) | |
| ECOG PS |  | 0.383 |  | 0.314 |
| 0-1 | 131/147(89.1) |  | 46/147(31.3) | |
| 2 | 3/4(75.0) |  | 0/4(0.0) | |
| Smoking history |  | 0.238 |  | 0.325 |
| Never | 51/60(85.0) |  | 21/60(35.0) | |
| Now/ever | 83/91(91.2) |  | 25/91(27.5) | |
| Distant metastasis |  | 0.124 |  | 0.588 |
| No | 31/32(96.9) |  | 11/32(34.4) | |
| Yes | 103/119(86.6) |  | 35/119(89.4) | |
| Degree of differentiation |  | 0.362 |  | 0.887 |
| Low | 101/116(87.1) |  | 35/116(30.2) | |
| Moderate/high | 33/35(94.3) |  | 11/35(31.4) | |
| Lines of therapy |  | 0.042 |  | 0.610 |
| 1 | 58/61(95.1) |  | 20/61(32.8) | |
| ＞1 | 76/90(84.4) |  | 26/90(28.9) | |
| Radiotherapy |  | 0.021 |  | 0.036 |
| No | 71/85(83.5) |  | 20/85(23.5) | |
| Yes | 63/66(95.5) |  | 26/66(39.4) | |
| NLR0W |  | 0.457 |  | 0.957 |
| ＞2.96  ≤2.96 | 68/75(90.7)  66/76(86.8) |  | 23/75(30.7)  23/76(30.3) | |
| PLR0W |  | 0.819 |  | 0.385 |
| ＞159  ≤159 | 67/75(89.3)  67/76(88.2) |  | 21/75(28.0)  25/76(32.9) | |
| PAR0W (*10^9) |  | 0.775 |  | 0.265 |
| ＞5.15  ≤5.15 | 66/75(88.0)  68/76(89.25) |  | 26/75(34.7)  20/76(26.3) | |
| Hb0W (g/L) |  | 0.423 |  | 0.086 |
| ≥130  ＜130 | 65/75(86.7)  69/76(90.8) |  | 18/75(24.0)  28/76(36.8) | |
| LDH0W (U/L) |  | 0.083 |  | 0.935 |
| ＞245  ≤245 | 34/42(81.0)  100/109(91.7) |  | 13/42(31.0)  33/109(30.3) | |
| CEA0W (ng/ml) |  | 0.621 |  | 0.093 |

| ＞3.5 | 87/97(89.7) |  | 25/97(25.8) |  |
| --- | --- | --- | --- | --- |
| ≤3.5 | 47/54(87.0) |  | 21/54(38.9) |  |
| NSE_0w_ (ng/ml)  ＞16.3  ≤16.3 |  | 0.248 |  | 0.320 |
|  | 64/72(88.9) |  | 24/72(33.3) |  |
|  | 66/70(94.3) |  | 18/70(25.7) |  |
| NLR6w  Up | 65/77(84.4) | 0.086 | 15/77(19.5) | 0.003 |
| Down PLR6w  Up | 69/74(93.2)  61/71(85.9) | 0.301 | 31/74(41.9)  20/71(28.2) | 0.564 |
| Down PAR6w  Up | 73/80(91.3)  58/67(86.6) | 0.450 | 26/80(32.5)  17/67(25.4) | 0.225 |
| Down  Hb6w Up | 76/84(90.5)  46/49(93.9) | 0.166 | 29/84(34.5)  17/49(34.7) | 0.434 |
| Down LDH6w  Up | 88/102(86.3)  68/81(84.0) | 0.045 | 29/102(28.4)  20/81(24.7) | 0.097 |
| Down CEA6w  Up | 66/70(94.3)  57/70(81.4) | 0.008 | 26/70(37.1)  17/70(24.3) | 0.125 |
| Down  NSE_6w_  UP  Down NLR12w  Up | 77/81(95.1)  46/51(90.2)  84/91(92.3)  67/77(87.0) | 0.664  0.027 | 29/81(35.8)  11/51(21.6)  31/91(34.1)  15/77(19.5) | 0.117  0.001 |
| Down PLR12w  Up | 67/69(97.1)  62/68(91.2) | 0.804 | 31/69(44.9)  21/68(30.9) | 0.879 |
| Down PAR12w  Up | 72/78(92.3)  49/55(89.1) | 0.369 | 25/78(32.1)  17/55(30.9) | 0.904 |
| Down  Hb12w Up | 85/91(93.4)  45/48(93.8) | 0.751 | 29/91(31.9)  15/48(31.3) | 0.963 |
| Down LDH12w  Up | 89/98(90.8)  73/82(89.0) | 0.170 | 31/98(31.6)  20/82(24.4) | 0.036 |
| Down CEA12w  Up | 61/64(95.3)  53/63(84.1) | 0.003 | 26/64(40.6)  13/63(20.6) | 0.014 |
| Down | 81/83(97.6) |  | 33/83(39.8) |  |
| NSE_6w_  UP  Down | 41/46(89.1)  88/95(92.3) | 0.485 | 12/46(26.1)  30/95(31.6) | 0.504 |

Up: elevated compared to pre-treatment Down: decreased compared to pre-treatment

Abbreviations: ECOG: Eastern Cooperative Oncology Group; PS: performance status; NLR: neutrophil-to- lymphocyte ratio; PLR: platelet-to-lymphocyte ratio; PAR: platelet-to-albumin ratio; Hb: hemoglobin; LDH: lactate dehydrogenase; CEA: carcinoembryonic antigen; NSE: neuron-specific enolase.
